# The Effects of Ghana’s National Health Insurance Scheme on Maternal and Infant Health Care Utilization

**DOI:** 10.1371/journal.pone.0165623

**Published:** 2016-11-11

**Authors:** Igna Bonfrer, Lyn Breebaart, Ellen Van de Poel

**Affiliations:** 1 Erasmus University Rotterdam, Institute for Health Policy & Management, Rotterdam, South Holland, The Netherlands; 2 Harvard University, Harvard T.H. Chan School of Public Health, Cambridge, Massachusetts, United States of America; INDEPTH Network, GHANA

## Abstract

Increasing equitable access to health care is a main challenge African policy makers are facing. The Ghanaian government implemented the National Health Insurance Scheme in 2004 and the aim of this study is to evaluate its early effects on maternal and infant healthcare use. We exploit data on births before and after the intervention and apply propensity score matching to limit the bias arising from self-selection into the health insurance. About forty percent of children had a mother who is enrolled in this insurance. The scheme significantly increased the proportion of pregnancies with at least four antenatal care visits with 7 percentage points and had a significant effect on attended deliveries (10 percentage points). Caesarean sections increased (6 percentage points) and the number of children born from an unwanted pregnancy decreased (7 percentage points). Insurance enrollment had almost no effect on child vaccinations. Among the poorest forty percent of the sample, the effects of the scheme on antenatal care and attended deliveries were similar. However, the effects of the scheme on caesarean sections were about half the size (3 percentage points) and the reduction in unwanted pregnancies was larger (10 percentage points) compared to the effects in the full sample. We conclude that in the first years of operation, the National Health Insurance Scheme had a modest impact on the use of antenatal and delivery care. This is important for other African countries currently introducing or considering a national health insurance as a means towards universal health coverage.

## Introduction

Increasing equitable access to health care is one of the main challenges African policy makers are facing today. An important constraint to healthcare access derives from the large out-of-pocket payments incurred at the point of use. Health insurance can protect households from the risk of medical expenses which can be large relative to modest incomes [1] and therefore cause households to fall into poverty [2]. The National Health Insurance Scheme (NHIS) implemented in Ghana in 2004 [3,4] is one of the most ambitious health care financing reforms in Sub Saharan Africa (SSA). Ghana has been the first country in SSA to establish a large financial protection scheme and other African countries working towards Universal Health Coverage are closely watching its progress [5]. The aim of this study is to estimate the nationwide effects of the NHIS on maternal and infant care utilization in its initial years of operation.

Previous research evaluated the early effects of the NHIS on health care use in specific geographic areas, i.e. the Accra Metropolitan Area [4] and the Brong-Ahafo and Upper East region [6]. Given that enrollment rates were highest in the latter two regions at the time of these studies [7], the findings from these two areas are not necessarily representative for other parts of Ghana. Using cross sectional data for propensity score matching (PSM) Blanchet et al. find that women enrolled in the NHIS are more likely to get prescriptions, visit clinics and seek formal care, but they do not study effects on maternal and infant health care [4]. Mensah et al. [6] also apply PSM and show that enrolled women in the Brong-Ahafo and the Upper East region are more likely to use antenatal care, deliver in a healthcare facility and are less likely to have birth complications. One unpublished study [8] did estimate the early nationwide effects of the NHIS. The authors exploit variations in the insurance design using an instrumental variable approach, and show that participation in the NHIS increases the probability of seeking curative and preventive care. However, they study a smaller set of outcomes, do not investigate differences in effects across the entire versus the poor population and use a different methodological approach.

Our work adds to the limited knowledge about the nationwide effects of the NHIS on maternal and infant health care utilization and specifically provides insights into the differences in effects by socioeconomic status. The latter is of particular importance given that the distributional impact of these schemes is one of the biggest knowledge gaps in the field [9].

### The National Health Insurance Scheme in Ghana

The National Health Insurance Scheme was implemented in 2004 to improve financial access, especially for the poorest and most vulnerable groups [10,11]. Membership of the NHIS is legally mandatory, but in practice membership is voluntary [12]. There is no penalty for failing to enroll and individuals are not automatically part of the scheme [4]. All children below 18 years old with parents or guardians covered under the NHIS can receive insured services for free after enrolling into a scheme and paying about 2 US$ for an insurance card. These children do not have to pay an insurance premium [8,13,14]. While enrollment rates for the NHIS might compare favorably to the low rates reported for smaller scale initiatives across SSA [15], low uptake remains a key problem and enrollment is far from universal [4,16,17]. Institutional data from the Ministry of Health and NHIS show an increase in average enrollment rates from 18 percent in 2006 to 55 percent in 2008 [18,19] and subsequently a decrease to 38 percent by the end of 2013 [20] which is partly caused by a new calculation method no longer looking at the total number of people who ever enrolled over time but only at active members. These figures are heavily debated [21], based on the Ghana Demographic and Health Survey (GDHS), 39.8% of recent mothers had an NHIS membership in 2008 and other studies suggest that about one third of the population was covered by 2011 [4,16,17]. Considerable socio-economic inequalities in enrollment exist [22]: better-off households are significantly more likely to enroll compared to the poorest quintile, with respective enrollment rates of 41 and 27 percent [23]. The low enrollment among the poor is likely to not only relate to lower economic well-being but also to considerable travel time to health facilities. Other authors suggest that fees could be too high for the poor [24], awareness about insurance low, or the perceived value for money insufficient [25,26]. In other words, for those who did not sign up, their perceived net benefits were negative, either because expected costs were too high or benefits were too low.

The NHIS is funded mainly through a value added tax on goods and services and further through social security taxes, individual premiums, investment returns and donors [4]. The benefit package is broad, covering more than 95 percent of conditions that afflict Ghanaians. This package consists of i) coverage of all costs, including food, associated with outpatient department and admission treatment, ii) full payment for medicine included in an approved list and iii) payments for referrals in an approved list [27].

Annual insurance premiums are income based, ranging from 7.2 Ghanain Cedi (GhC) (2.25 United States (US) $) to 48 GhC (14.85 US $). However, given unavailability of income measures, a constant premium of 3 US dollars is charged in most cases [4,28]. Exemptions nullifying the premium exist for the indigent [29] and for people over age 70. Prior to the implementation of the NHIS, in 2003, the Ghanaian government introduced a delivery fee exemption policy using a stepwise geographical roll-out leading to theoretical nationwide coverage in April 2005, i.e. before our study period and therefore unlikely to bias our estimates based on differences between insured and comparable uninsured. In practice the implementation did not have adequate financial backing and a system of standardized charging was not applied. Failure of prompt and adequate reimbursement to the facilities led to near failure of the policy [30]. During our study period, the early years of the NHIS, delivery care was part of the benefit package. In principle delivery was already free but in practice this was probably not always the case, this might have changed with the introduction of the NHIS, potentially compensating providers more adequately.

A few years after the introduction of the NHIS, the so called Maternal Exemption Policy was introduced. This policy aimed to abolish premiums for pregnant women and to automatically cover newborns to avoid creating a gap between the time of birth and the registration of the newborn. The program was introduced in some areas in late 2008 but only later in other areas. The program suffered from low awareness, limited reimbursement to the facilities and no monitoring of the implementation [31], making it difficult to determine who received an exemption and who did not. For more details on the scheme refer to Gobah & Zang [11] and Witter & Garshong [12].

## Materials and Methods

### Data

We use the GDHS 2008, a nationally representative household survey with individual data collected for 4916 women aged 15–49 [7]. These women were asked about all children born to them in the last five years (2003–2008) and more detailed information was obtained about the most recent live birth. We analyze these data at the level of the child and have information about every pregnancy preceding the birth of that specific child for the period 2006–2008. Even though data are analysed at the child level, all information is based on reports from their mothers. More recent data are available but these were collected after the introduction of the Maternal Exemption Policy that abolished insurance premiums for some pregnant women, though it is not exactly clear which women were reached with the program, and therefore does not allow for an identification of the effect of the NHIS as initially implemented.

Information about the NHIS enrollment status is available for the mother at the time of the interview, not at the time of pregnancy. This means we have to assume that enrollment status at time of interview is representative for the enrollment status during pregnancy. We limit our analysis to the most recent live birth, up to two years preceding the interview, to increase credibility of this assumption. This results in a sample of 2002 children from 1959 mothers (43 mothers were pregnant with twins), with a mean duration between birth and interview of 11 months. Further reduction to a period of one year preceding the interview, results in a sample too small to perform our analyses. Mensah et al. [6], make the same assumption but include births up to four years prior to the survey.

We study eight main outcomes: at least 4 antenatal care (ANC) visits, at least 4 ANC visits with a skilled provider (doctor, nurse/midwife, auxiliary midwife or community nurse), delivery assisted by a skilled provider, delivery assisted by a skilled provider in a public facility, caesarean section, pregnancy too soon or unwanted (occurred earlier than desired or when no or no more children were desired), child received vitamin A and child fully vaccinated (see Table 1). We differentiate between any assisted delivery and assisted delivery in a public facility to identify any switches from private to public facilities or from unattended homebirth to a public facility. Information about whether a child received vitamin A and the relevant vaccinations was based on the information recorded on the vaccination card. So if the card was missing or incomplete, no vitamin A or vaccination(s) were listed for the child in the GDHS data. The outcome “child fully vaccinated” was 1 if five relevant vaccinations (see below) were provided and 0 otherwise. Vaccinations provided at some stage after birth were calculated on the subsample of children born at least one year prior to the survey, reflecting that these vaccinations are obtained over the course of the first life year.

**Table 1:** Descriptives.

	*Full sample*			*Among the poor*		
	Uninsured	Insured	p-value	Uninsured	Insured	p-value
At least 4 ANC visits	0.80	0.91	0.000	0.76	0.85	0.000
At least 4 ANC visits, skilled provider	0.37	0.55	0.000	0.30	0.44	0.000
Attended delivery	0.40	0.71	0.000	0.25	0.46	0.000
Attended delivery in public facility	0.31	0.62	0.000	0.20	0.42	0.000
Caesarean section	0.03	0.10	0.000	0.01	0.04	0.009
Pregnancy too soon or unwanted	0.40	0.31	0.000	0.38	0.31	0.027
Child received vitamin A	0.48	0.58	0.001	0.48	0.59	0.008
Child fully vaccinated	0.37	0.53	0.000	0.30	0.40	0.010

Note: p-values shown for individual t-tests comparing means for uninsured (n = 1206) and insured (n = 796).

p-values shown for individual t-tests comparing means among the poor for uninsured (n = 804) and insured (n = 324).

Based on data from pregnancies and births in 2006–2008 collected through the Ghana Demographic and Health Survey 2008.

The pregnancy too soon or unwanted does not directly relate to services covered by the health insurance (family planning services are not covered under the NHIS) but could reflect potential spill-over effects i.e. family planning advice and services provided alongside maternal care. To our knowledge there were no nationwide, continuous initiatives before the implementation of the NHIS to provide free family planning services. Child vaccinations were not covered under the NHIS, there was a program for free child vaccinations from the Global Alliance for Vaccines and Immunization [32] organized jointly with the Ghanian government. We hypothesize to find no effect of the NHIS on infant vaccinations and use this outcome as a robustness measure to determine whether we indeed find no effect on this outcome. To provide additional insights into the details of the child vaccinations, we also present five additional outcomes: polio vaccination at birth, Bacillus Calmette-Guérin (BCG) vaccination, three additional polio vaccinations, three Diphtheria, Tetanus and Poliomyelitis (DTP) vaccinations and measles vaccination. These details on child vaccinations are provided as Supplemental Information, see S1 Table: Descriptives.

### Statistical analysis

Although NHIS membership is de jure compulsory, in practice enrollment is an individual’s choice. Comparing health care use between enrollees and non-enrollees can provide biased estimates of the effects of NHIS because it is likely that NHIS membership is driven by factors that also correlate with health and health care use. We therefore apply PSM to identify the control observations (non NHIS members i.e. uninsured) that are similar to the treated (NHIS members i.e. insured) in terms of observable characteristics [33]. Covariates used for the PSM are related to the mother’s demographics, socio-economic status, education, occupation, religion, ethnicity, region of residence and time period. We proxy socioeconomic status with the wealth index provided in the GDHS which is estimated through principal component analysis on a large set of assets and dwelling characteristics. [34]. Table 2 provides an overview of the covariates.

**Table 2:** Descriptive statistics and propensity score logit.

	Unmatched means		Differences	Propensity score logit (n = 2002)	
	Uninsured	Insured	p-value	Av. marg. effect	p-value
Rural household	0.76	0.56	0.000	Ref.	Ref.
Urban household	0.24	0.44	0.000	-0.01	0.651
Mother below 20y	0.06	0.03	0.005	Ref.	Ref.
Mother 20y–35y	0.76	0.79	0.088	0.11	0.034
Mother above 35y	0.18	0.18	0.797	0.18	0.003
Mother not married or living together	0.09	0.06	0.010	Ref.	Ref.
Mother married or living together	0.91	0.94	0.010	0.07	0.082
Mother's religion none or other	0.12	0.06	0.000	Ref.	Ref.
Mother's religion Christian	0.42	0.50	0.001	0.03	0.261
Mother's religion Muslim	0.21	0.21	0.928	0.01	0.706
Mother's ethnicity Akan	0.36	0.38	0.257	-0.11	0.002
Mothers' ethnicity Ewe	0.13	0.11	0.263	-0.15	0.001
Mother's ethnicity Mole-Dagbani	0.26	0.26	0.865	-0.07	0.035
Mother is illiterate	0.80	0.61	0.000	Ref.	Ref.
Mother is literate	0.20	0.39	0.000	-0.01	0.821
Mother secondary or higher education	0.27	0.52	0.000	Ref.	Ref.
Mother no or primary education	0.73	0.48	0.000	-0.18	0.000
Mother's occupation blue-collar	0.38	0.59	0.000	Ref.	Ref.
Mother's occupation none	0.11	0.10	0.800	-0.07	0.045
Mother's occupation white-collar	0.01	0.05	0.000	0.06	0.351
Mother's occupation agriculture	0.50	0.25	0.000	-0.10	0.000
Log household size	1.72	1.65	0.001	0.00	0.904
Household very poor	0.43	0.20	0.000	-0.40	0.000
Household poor	0.23	0.21	0.176	-0.20	0.000
Household moderate wealth	0.16	0.17	0.641	-0.17	0.000
Household rich	0.11	0.24	0.000	Ref.	Ref.
Household very rich	0.06	0.18	0.000	0.08	0.056
Ashanti	0.14	0.15	0.526	0.19	0.000
Brong-Ahafo	0.06	0.10	0.003	0.40	0.000
Central	0.10	0.04	0.000	Ref.	Ref.
Eastern	0.07	0.12	0.000	0.38	0.000
Greater Accra	0.09	0.08	0.291	-0.05	0.404
Northern	0.21	0.11	0.000	0.27	0.000
Upper East	0.05	0.11	0.000	0.60	0.000
Upper West	0.10	0.12	0.107	0.44	0.000
Volta	0.09	0.08	0.845	0.29	0.000
Western	0.10	0.09	0.858	0.22	0.000

Note: p-values shown for individual t-tests comparing means for uninsured (n = 1206) and insured (n = 796).

All models include time controls as well as the control variables listed above.

Ref. indicates that a variable is a reference variable.

Based on data from pregnancies and births in 2006–2008 collected through the Ghana Demographic and Health Survey 2008.

To obtain propensity scores for each respondent, we estimate a logit model because of the dichotomous outcome indicator child’s mother is enrolled in the NHIS or not. All covariates used in this regression model are shown in Table 2. We also include indicators for each birth quarter (three month period) within the time period in our data to pick up any time-varying characteristics. For ease of interpretation, we report average marginal effects as opposed to coefficients.

We apply Kernel matching to match the children from insured mothers to comparable controls on the basis of their propensity scores. Kernel matching constructs a hypothetical match for each treated observation using a weighted average of all controls. The weights are determined by the inverse of the distance to the propensity score of the child. We use the routine “psmatch2” in the statistical software package STATA 13 by Leuven and Sianesi [35] to calculate the average treatment effect on the treated (ATT). As a robustness check we apply three other matching algorithms: nearest neighbour with replacement, nearest neighbour without replacement and radius matching. A detailed description of these algorithms can be found in Khandker et al. [36] and results from the robustness checks are available upon request from the authors.

**Fig 1:**
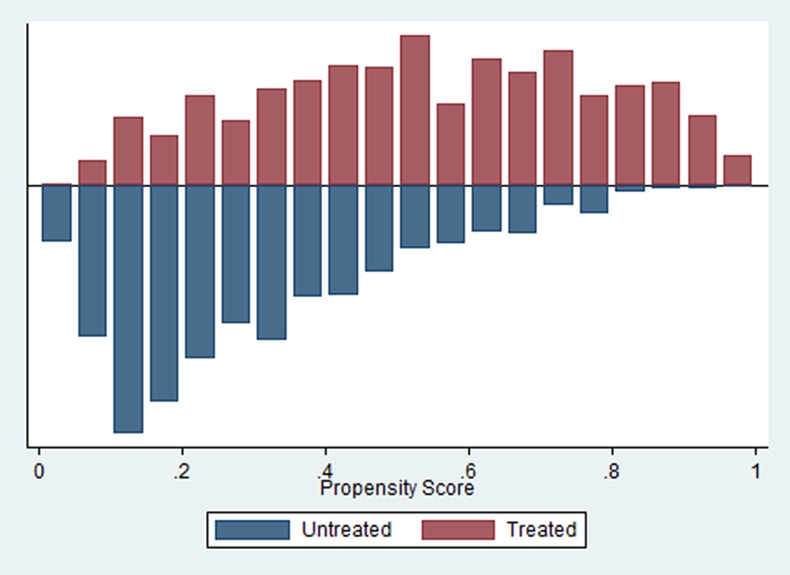
Propensity scores across untreated (matched control observations) and treated.

We conduct a balancing test to check whether, within each quantile of the propensity score distribution, both the average propensity score and the mean of the explanatory variables are the same between the treated and control individuals. This ensures that the treated and the matched controls are balanced in that similar propensity scores are based on similar explanatory variables [37]. Fig 1 shows the propensity scores for both the treated and the untreated.

The validity of the PSM approach generally depends on two conditions: i) conditional independence and ii) sizeable common support or overlap in propensity scores across the treated and the matched control sample [36,38]. The first, requiring no unobserved characteristics to affect the decision to enroll cannot be tested. However, our matching on extensive information on maternal, household, child, regional and time characteristics, including socio-economic status, should eliminate most of the important drivers of selection bias, though some bias due to unobserved differences is likely to remain. Ensuring validity of PSM in relation to the second condition is done in line with general practice by only considering observations on the common support of the propensity scores across the treated and the matched controls [36]. Following Leuven and Sianesi [35], any observations with a propensity score higher than the maximum or lower than the minimum score of the controls are dropped, which results in a loss of less than one percent of our sample.

### Effects among the poor

**Fig 2:**
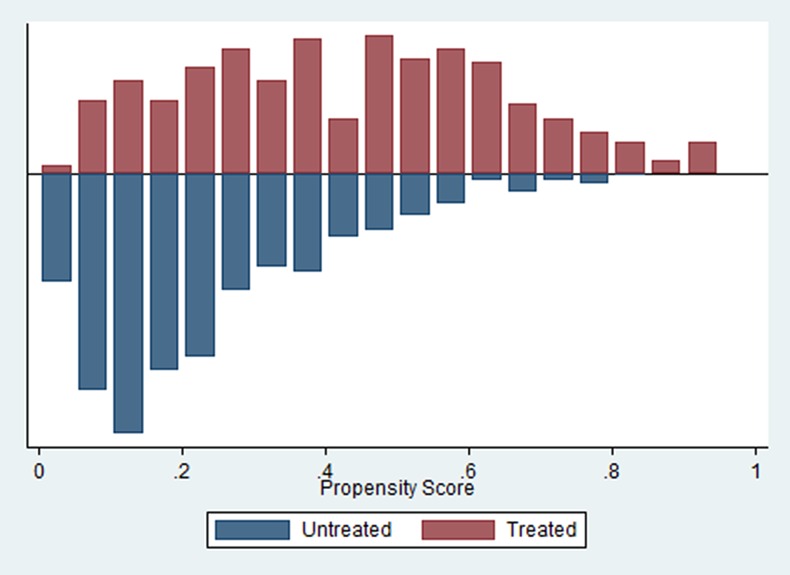
Propensity scores across untreated (matched control observations) and treated among the poor.

NHIS premiums might be high for the poorer segments of the population and costs associated with health care use not covered by the NHIS, such as travel costs, might prove to be a considerable financial burden to the less well-off. This potentially results in lower maternal and infant care utilization among the forty percent poorest share of respondents (the bottom two wealth quintiles) which we refer to as the “poor” from this point onward. We therefore study the heterogeneity in the effects of the NHIS across the poor, compared to the entire study sample. We re-estimate the propensity scores and apply the PSM only on the subsample of poor respondents. For consistency reasons we use the same decision-to-enroll model as applied for the full sample. Balance was achieved on all covariates apart from Mother’s religion Muslim and Mother’s ethnicity Mole-Dagbani. The same propensity score matching method is applied to contrast the average outcomes between the poor members and the poor non-members. Fig 2 shows the propensity scores across the poor for both the treated (NHIS members) and the untreated (non NHIS members).

## Results

### Descriptive statistics

Table 1 shows that for all types of health care utilization included in this study, the use is significantly higher among the insured (p ≤ 0.001). The prevalence of too soon or unwanted pregnancies is significantly lower among the insured (40 versus 31 percent, p < 0.000). Details about the different vaccinations are provided in S1 Table: Descriptives.

39.8% of the children in our sample have a mother who is insured. Table 2 shows the unmatched means for the covariates across the insured and uninsured group. Urban residence, the mother being married, being Christian, being literate, being in white collar occupation and the household being in the upper wealth quintile are all more common among those with insurance. Mothers with no or only primary education, working in agriculture, with larger households and from poor households are significantly less common among the insured. Further, living in the Brong-Ahafo and the Upper East region is more common among those with insurance.

### Correlates of NHIS enrollment

The last two columns of Table 2 contain the results of a logit model showing the correlates of participation in the NHIS. Average marginal effects for mother, household, child and regional characteristics are presented, time indicators are omitted for the sake of parsimony. The age of the child’s mother, marital status, ethnicity, education, occupation, wealth and region of residence are important predictors of the decision to enroll in the NHIS. The probability that a 20 to 35 years old mother enrolls in the NHIS is 11 percentage points (pp) higher (p = 0.034) compared to younger women and for mothers above 35 years old this probability is even 18 pp higher (p = 0.003). The probability that a mother who is married or living together is enrolled is 7 pp higher (p = 0.082), while mothers from the three largest ethnic groups in our sample: Akan, Ewe or Mole-Dagbani are significantly less likely to enroll compared to those from the minority ethnic groups (including Ga-Adang me, Guan, Grusi, Gruma and Mande). Mothers with no or only primary education have an 18 pp lower probability (p = 0.000) to enroll and those without occupation (7 pp, p = 0.045) or working in agriculture (10 pp, p = 0.000) are also less likely to enroll. The probability to enroll increases with household wealth. Compared to children whose mothers are residing in Central region, enrollment is significantly more likely in all other regions but Great Accra. In the following step of our analysis, we use this logit model as the principal mean to construct a balanced sample of NHIS members and comparable non NHIS members.

### Effects of NHIS enrolment

Having verified that the balancing property is satisfied, we present the ATT of being insured on maternal and infant health care use. Table 3 shows that NHIS enrollment increased the percentage of children who obtained at least four ANC visits by 7 percentage points (pp) (p = 0.009). When looking specifically at four or more ANC visits provided by skilled providers, the effect is smaller and no longer statistically significant. NHIS membership has a positive effect on attended delivery (10 pp, p = 0.002). When limiting this to only include the attended deliveries in public facilities, we again find significant positive effects of NHIS enrollment, with 12 pp increase (p = 0.000), which suggests that NHIS membership is not so much crowding out private deliveries. Caesarean sections increased with 6 pp (p = 0.000). The number of children born from a pregnancy which was too soon or unwanted is 7 pp lower (p = 0.039) among insured. NHIS membership seems to have had no effect on children receiving Vitamin A supplements, nor on childhood vaccinations (see S2 Table: Effects of insurance), as we expected because these services were not covered by the NHIS and largely provided for free. These findings are robust to the use of different matching algorithms; results are available upon request from the authors.

**Table 3:** Effects of insurance.

	Unmatched difference	Kernel^a^	p-value
At least 4 ANC visits	0.11	0.07	0.009
At least 4 ANC visits, skilled provider	0.18	0.05	0.114
Attended delivery	0.31	0.10	0.002
Attended delivery in public facility	0.31	0.12	0.000
Caesarean section	0.07	0.06	0.000
Pregnancy too soon or unwanted	-0.09	-0.07	0.039
Child received vitamin A	0.10	0.08	0.106
Child fully vaccinated	0.16	0.06	0.212

Effects are shown for Kernel matching, results for other matching methods (nearest neighbor with replacement, nearest neighbor without replacement and radius) are available upon request from the authors. Values shown are the difference between the average treatement effect on treated for uninsured and insured.

This model includes time controls as well as the control variables listed in Table 2.

Based on data from pregnancies and births in 2006–2008 collected through the Ghana Demographic and Health Survey 2008.

a Chosen bandwith: 0.06

### Effects of NHIS enrollment among the poor

To study the effects of NHIS enrollment among lower socio-economic groups, we estimated the effects among the children from poor households (for sample sizes see Table 1). A comparison of means before matching shows significantly higher utilization rates among the insured and a lower rate of children born from pregnancies which were too soon or unwanted (see Table 4).

**Table 4:** Effects of insurance among the poor.

	Unmatched difference	Kernel^a^	p-value
At least 4 ANC visits	-0.09	0.07	0.027
At least 4 ANC visits, skilled provider	-0.14	0.05	0.171
Attended delivery	-0.21	0.11	0.003
Attended delivery in public facility	-0.22	0.12	0.001
Caesarean section	-0.02	0.03	0.023
Pregnancy too soon or unwanted	0.10	-0.10	0.009
Child received vitamin A	-0.10	0.05	0.368
Child fully vaccinated	-0.09	0.04	0.401

Note: p-values shown for effects on outcome measures.

Effects are shown for Kernel matching, results for other matching methods (nearest neighbor with replacement, nearest neighbor without replacement and radius) are available upon request from the authors. Values shown are the difference between the average treatement effect on treated for uninsured and insured.

The model includes time controls as well as the control variables listed in Table 2.

Based on data from pregnancies and births in 2006–2008 collected through the Ghana Demographic and Health Survey 2008.

^a^ Chosen bandwith: 0.06

Results from the PSM reveal that NHIS membership had a similar positive effect on ANC visits (7 pp, p = 0.027) for the poor. We find significant effects on attended deliveries as a result of NHIS enrollment (11 pp, p = 0.003). Caesarean sections also increased significantly with 3 pp (p = 0.023), which is half of the effect in the full sample. NHIS membership reduced unwanted pregnancies among the poor (10 pp, p = 0.009), which is a larger reduction than in the full sample (7 pp). No effect was found on vitamin A supplements or on child vaccinations (see also S3 Table: Effects of insurance among the poor), which is in line with the findings from the full sample.

## Discussion

We compared maternal and infant health care utilization between children from mothers with and without enrollment in the National Health Insurance Scheme (NHIS) in Ghana. We used propensity score matching (PSM) to limit the potential bias arising from the self-selection into the NHIS. 39.8% of the children have a mother who is enrolled in the NHIS and the mother’s age, marital status, ethnicity, education, occupation, wealth and region of residence are the main correlates of enrolment. We found that NHIS enrollment significantly increased the percentage of children whose mother obtained at least four antenatal care (ANC) visits, had a skilled health care worker present during birth and that were born with a caesarean section. The average caesarean section rate for NHIS members, 10 percent, is just in the range advised by the World Health Organization of 10 to 15 percent [39], which suggests that the NHIS does not over-incentivize providers to perform caesarian sections. NHIS had no effect on services not covered by the insurance (vitamin A supplements and child vaccinations), which is in line with our expectations. We found little difference in effects across the poor compared to the full sample. The reduction in unwanted pregnancies is particularly large among the poor.

Our results are generally in line with, but smaller than, the study by Mensah et al. [6] who found in the Brong-Ahafo and Upper East region increases in ANC utilization of 20 to 23 pp, compared to the 7 pp in our study. The difference might be partly driven by the fact that we studied at least four ANC visits while Mensah et al. used at least three ANC visits, and to selectiveness of their sample. The effects on attended delivery in our study (11 pp increase) are similar to the 14 to 16 pp increase that Mensah et al. report. Both studies found no effect on polio vaccinations.

Providing that our results from the initial years of the NHIS hold up as the coverage is extended, increased enrollment in the NHIS leads to modest improvements in maternal health care utilization. We also found a significantly lower rate of pregnancies reported as too soon or unwanted among the insured, even though family planning services are not covered by the NHIS. This might be caused by spill-over effects i.e. family planning advice and services provided alongside maternal and infant care or a general increased awareness about the availability of health care services, including family planning. More qualitative research would be necessary to determine the factors driving the reduction of unwanted pregnancies among insured women.

Following the period studied here, in late 2008, the Maternal Exemption Policy was implemented for some pregnant women [13,14]. We do not have the necessary data to study the effects of this intervention, but given the socio-economic inequality in both enrollment and to some extent maternal and infant care utilization, alleviating the financial burden of premium payments might have been especially effective in increasing access for the poor segment of the population. Our results therefore provide a lower bound for the effects of the current NHIS on maternal and infant care utilization.

Our analyses are subject to some limitations. First, the NHIS rollout was non-randomized, we control for self-selection into the NHIS on observables but there might be remaining bias due to unobserved differences across the member and the matched non-member group that also correlate with health care use. Adverse selection should be muted by the required waiting period of a maximum of six months [40], usually three months [27] after enrollment. A second limitation is related to the assumption that women’s enrollment status at the date of the interview is representative for their status during their pregnancy in the last two years. Given that enrolment rates were increasing over time in this period [18], we are likely to overestimate NHIS enrolment status at birth/pregnancy and therefore underestimate the effect of NHIS membership on maternal and infant health care use. Third, while the GDHS is nationally representative at the time of survey, it is not necessarily so for the sample of births which took place in only the two years previous to the survey. Fourth, before the introduction of the NHIS there was some provision of free vaccinations by the Global Alliance for Vaccines and Immunization which might partly explain the limited effects we found. Because of data limitations we are not able to determine when and where these vaccinations were provided to study this further. Finally, we evaluate the effects of the NHIS only in its early years. Over time the scheme has changed, most notably through the abolition of premiums for pregnant women. Nevertheless, our results are of importance to other sub Saharan African countries currently considering the rollout of a national health insurance scheme.

## Supporting Information

S1 Table Descriptives. (DOCX)

S2 Table Effects of insurance. (DOCX)

S3 Table Effects of insurance among the poor. (DOCX)
