# Supplementary material for: The Effects of Ghana’s National Health Insurance Scheme on Maternal and Infant Health Care Utilization
Source: PLoS One. 2016 Nov 11;11(11):e0165623. doi: 10.1371/journal.pone.0165623 (PMC5106190; doi:10.1371/journal.pone.0165623)
Supplement: Supplementary file 1 [file pone.0165623.s001.docx]

S1 Table: Descriptives

	Full sample			*Among the poor*		
	**Uninsured**	**Insured**	**p-value**	**Uninsured**	**Insured**	**p-value**
Child received polio vaccination at birth	0.39	0.55	0.000	0.32	0.43	0.001
Child received BCG vaccination	0.68	0.78	0.001	0.66	0.74	0.051
Child received all three polio vaccinations	0.65	0.76	0.000	0.64	0.71	0.072
Child received all three DTP vaccinations	0.67	0.76	0.001	0.66	0.72	0.105
Child received measles vaccination	0.66	0.77	0.000	0.65	0.73	0.035

Note: p-values shown for individual t-tests comparing means for uninsured (n = 736) and insured (n = 440).
p-values showns for individual t-tests comparing means among the poor for uninsured (n = 493) and insured (n = 196).
Based on data from pregnancies and births in 2006-2008 collected through the Ghana Demographic and Health Survey 2008.
