# Supplementary material for: The Effects of Ghana’s National Health Insurance Scheme on Maternal and Infant Health Care Utilization
Source: PLoS One. 2016 Nov 11;11(11):e0165623. doi: 10.1371/journal.pone.0165623 (PMC5106190; doi:10.1371/journal.pone.0165623)
Supplement: Supplementary file 2 [file pone.0165623.s002.docx]

S2 Table: Effects of insurance

	**Unmatched difference**	**Kernel**[a]	**p-value**
Child received polio vaccination at birth	0.16	0.05	0.147
Child received BCG vaccination	0.10	0.03	0.478
Child received all three polio vaccinations	0.11	0.04	0.424
Child received all three DTP vaccinations	0.09	0.03	0.522
Child received measles vaccination	0.11	0.09	0.045

Note: p-values shown for effects on outcome measures.

Effects are shown for Kernel matching, results for other matching methods (nearest neighbor with replacement, nearest neighbor without replacement and radius) are available upon request from the authors. Values shown are the difference between the average treatement effect on treated for uninsured and insured.

This model includes time controls as well as the control variables listed in Table 2.

Based on data from pregnancies and births in 2006-2008 collected through the Ghana Demographic and Health Survey 2008.

[a] Chosen bandwith: 0.06
